# Conspecific and Heterospecific Cues Override Resource Quality to Influence Offspring Production

**DOI:** 10.1371/journal.pone.0070268

**Published:** 2013-07-08

**Authors:** Christine W. Miller, Robert J. Fletcher, Stephanie R. Gillespie

**Affiliations:** 1 Department of Entomology and Nematology, University of Florida, Gainesville, Florida, United States of America; 2 Department of Wildlife Ecology and Conservation, University of Florida, Gainesville, Florida, United States of America; Fundação Oswaldo Cruz, Brazil

## Abstract

Animals live in an uncertain world. To reduce uncertainty, animals use cues that can encode diverse information regarding habitat quality, including both non-social and social cues. While it is increasingly appreciated that the sources of potential information are vast, our understanding of how individuals integrate different types of cues to guide decision-making remains limited. We experimentally manipulated both resource quality (presence/absence of cactus fruit) and social cues (conspecific juveniles, heterospecific juveniles, no juveniles) for a cactus-feeding insect, *Narnia* *femorata* (Hemiptera: Coreidae), to ask how individuals responded to resource quality in the presence or absence of social cues. Cactus with fruit is a high-quality environment for juvenile development, and indeed we found that females laid 56% more eggs when cactus fruit was present versus when it was absent. However, when conspecific or heterospecific juveniles were present, the effects of resource quality on egg numbers vanished. Overall, *N* *. femorata* laid approximately twice as many eggs in the presence of heterospecifics than alone or in the presence of conspecifics. Our results suggest that the presence of both conspecific and heterospecific social cues can disrupt responses of individuals to environmental gradients in resource quality.

## Introduction

Animals often live in variable and uncertain environments. To increase survival and reproductive success, individuals must continually gather information on their surroundings through cues about the environment. Such information can be diverse, ranging from simple cues of food availability to more complex cues regarding the quality of potential mates [1–4]. This diversity of cues and the information encoded in them has received a great deal of interest in recent years, with a common distinction being cues obtained from the behavior of other individuals, termed “social information”, to those obtained directly from the environment, termed “non-social information” [5]. Several models suggest that using information derived from social cues may provide efficient means to gauge the quality of a particular area [6–9]. Understanding the use of social cues in behavioral decision-making can help explain a variety of patterns observed in animals, ranging from social aggregations to species distributions across broad spatial scales [10,11].

While social information can help animals make crucial decisions, it is likely used in conjunction with information that does not come from other individuals in the environment [12]. Some studies have shown that decision making may be dependent upon the consistency between social and non-social cues [12–14], whereas others suggest that some cues can effectively trump others [15–18]. However, our understanding of how individuals weigh different cues in decision-making and the potential for context-dependence use of cues remains limited.

The source of social information is typically assumed to arise from conspecifics [19]. While individuals may often rely on social information from conspecifics, it is also possible that relevant heterospecific cues may be used [3,20]. Using heterospecific information to make important life-history decisions may be more effective than using conspecific information in some situations, and the use of this information can have consequences for community ecology at different scales [21–23]. In particular, heterospecifics may be weaker competitors than conspecifics, but yet may also share many habitat requirements [3]. Therefore, decision-making based on the presence of heterospecifics versus conspecifics may yield many of the benefits of social information use, but fewer of the costs. Although there has been increasing interest in understanding the role of conspecific versus heterospecific cues in decision-making, studies are still rare. Comparing and contrasting the use of social cues with non-social cues is essential for understanding how information is used in a community context.

**Figure 1:**
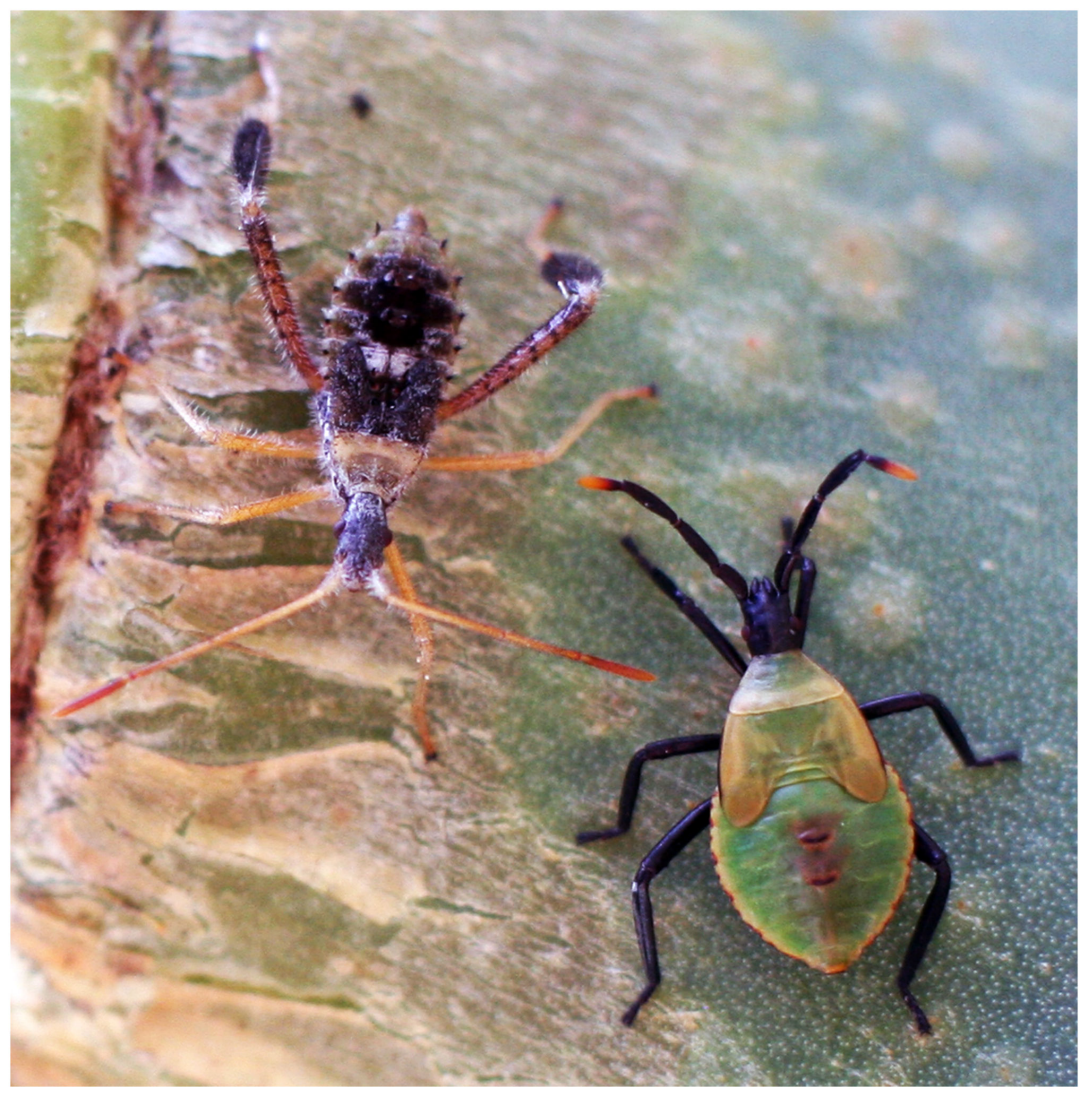
Juvenile *Narnia* *femorata* (left) and *Chelinidea* *vittiger* (right) on the same *Opuntia* *cactus* . Juveniles form aggregations that can include members of both species.

Here, we tested the extent to which individuals respond to conspecifics and heterospecifics in environments of differing resource quality. *Narnia* *femorata* and *Chelinidea* *vittiger* , two species of cactus-feeding bugs, form both within- and across-species aggregations on cactus patches ( *Opuntia* and *Cylindropuntia* *spp.*) [24]. They are close relatives, both in the family of leaf-footed bugs (Hemiptera: Coreidae). The similar size and morphology of *N* *. femorata* and *C* *. vittiger* juveniles (Figure 1) as well as direct observations suggest that they share common predators, primarily arthropods such as spiders and ants (Miller, *personal observation*). They both feed on cacti; however, these two species prioritize different parts of cactus plants for feeding. *C* *. vittiger* individuals feed shallowly on cactus pads ( *Cladodes* ), while *N* *. femorata* use their longer mouthparts to feed on deep tissue and seeds in cactus fruits. The presence of cactus fruit is important for *N* *. femorata* development rate, adult size, and mating success [25], yet *C* *. vittiger* does not require cactus fruits. Thus, *C* *. vittiger* and *N* *. femorata* share similarities and differences in habitat requirements, presenting excellent opportunities to examine the roles of social and non-social information in a community context.

We exposed *N* *. femorata* adult females to cactus with and without fruit and the presence or absence of conspecific and heterospecific juveniles to examine the role of social and non-social cues on the number of eggs laid. In the wild, female *N* *. femorata* move frequently among cactus patches and will lay variable-sized clutches on different patches. Juveniles are wingless and limited in mobility; thus, female egg numbers largely determine the number of offspring that will experience a given habitat. Juveniles of both species preferentially aggregate, and mixed-species aggregations are common (Figure 1). Previous work has demonstrated that the presence of juveniles or eggs on a cactus patch increases offspring production in the heterospecific, *C* *. vittiger* [26]. We predicted that female *N* *. femorata* would lay greater numbers of eggs in the presence of the high-quality resource (cactus with ripe fruit) [25]. Furthermore, we predicted the presence of *C* *. vittiger* and *N* *. femorata* juveniles would both boost the number of eggs laid, with the greatest numbers of eggs laid in the presence of the heterospecific.

## Materials and Methods

### Insect rearing

*Narnia* *femorata* used in this experiment were third and fourth generation laboratory animals. The original lab population was established from fall 2009 field collections at the University of Florida, Ordway-Swisher Biological Station (29° 41' N, 82° W). In fall 2010, *N* *. femorata* were reared with *O* *. humifusa* Cladodes and fruit in family groups of 4-8 individuals in plastic containers [26] to mimic natural aggregative behaviors seen in young juveniles in the wild. All subjects came from a total of 40 parent pairs and were kept in a 10-30 °C greenhouse with 14-hours of light/day. We separated out older juveniles into individual containers, allowing us to keep newly molted adults unmated until the testing period.

*Chelinidea* *vittiger* used in this experiment were the offspring of adult males and females collected from the UF Ordway-Swisher Biological Station. These adult males and females were paired, and their young offspring were reared on cactus pads without fruits at natural densities ( *C* *. vittiger* typically lays 3-8 egg clutches in this area [24]) in plastic containers until the second or third instar, when they were used in the experiment (see below).

### Experimental design

Our experimental design focused on manipulating putative cues likely to be used in decision-making by *N* *. femorata* . We manipulated both the social environment and resource quality to determine the extent to which they resulted in the alteration of *N* *. femorata* oviposition. All females used in this experiment were reared with similar food, cactus pads and late-season green fruits, because previous work suggested that rearing environment of *N* *. femorata* females can influence adult mating behavior and oviposition (Miller, *unpublished data*). Males in this experiment were reared during their final two instars on cactus without or with red and green cactus fruits, reflecting the natural range of resource quality in the fall when cactus fruits turn from green to red and many are rapidly removed from the plants by other herbivores [Gillespie, *unpublished data*]. Females were randomly paired with unrelated males when both were 14-21 days of age. We ensured males from all rearing environments were spread across all environments (below). We also made attempts to spread family members across treatments to minimize the chance of bias. We placed male-female pairs in one of six factorial combinations of social and non-social environments. Non-social environments included either a cactus cladode, or a cactus cladode and an attached red fruit. Social environments included five second or third instar *Chelinidea* *vittiger* juveniles, five second or third instar *N* *. femorata* juveniles, or no juveniles. Our design reduced the likelihood that there was an added cost of acquiring non-social cues relative to social cues [11] because both cues, nymphs and fruit, were readily available. The pairs were left together for seven days, each in one of six spatial blocks within a greenhouse. We did not anticipate a reduction in resource quality during this time period because we have frequently witnessed groups of this size feeding on cactus plants for multiple weeks in the wild, with only minor visible damage. We checked pairs every 48 hours and recorded any deaths. We did not use data from pairs where a member of the pair died during the seven day experiment. On the eighth day, we counted all eggs and froze adults for later morphometric analysis.

### Morphometrics

We photographed each *Narnia* *femorata* female to quantify body size, because size is a strong predictor of fecundity in invertebrates. Pictures were taken with a Canon EOS 50D camera and Leica M 165C microscope. The insects were measured using ImageJ 1.42q software. Length measurements (in mm) were taken on the head, hind femurs (left and right), and front femurs (left and right). Width measurements (in mm) were taken on the pronotum and hind femurs (left and right). We found the area (in mm^2^) of the hind femurs (left and right) and hind tibias (left and right) using the Image-J threshold function. We then averaged all of the left and right leg measurements. These seven measurements were then used in a Principal Component Analysis (PCA) to determine a composite body size index (first principal component) of each individual.

### Statistical analyses

We used a generalized linear mixed model (GLMM) to test whether social environment (conspecifics, heterospecifics, or no individuals), cactus quality (with or without fruit) and their interaction influenced oviposition by females. Because the number of eggs produced is count data, a Poisson distribution was a natural choice for this GLMM [24]; however, a model assuming a Poisson error distribution was overdispersed. We considered using a negative binomial error distribution but that model did not converge. We therefore used a generalized Poisson error distribution instead that accounts for overdispersion (along with a log link function), which is appropriate when response data show strong, right-skewed distributions, as in our case (see below) [27]. Block (i.e. greenhouse location) was included as a random effect. We controlled for female size by including the first principal component derived from a PCA as a nuisance covariate (see *Morphometrics* above). Note that we checked for heterogeneity of regression slopes by including pair-wise interactions of PC1 with social environment and cactus quality, but found no evidence for such effects (PC1*social: *F*_2,132_ = 1.14, *P* = 0.32; PC1*cactus: *F*_1,132_ = 2.31, *P* = 0.13) so the final model did not include these interactions. Prior to the final analysis, we also considered whether the rearing environment of males influenced female oviposition. We therefore initially included male rearing environment and its pair-wise interaction with cactus quality and social environment as nuisance covariates. We found no evidence for male rearing environment influencing oviposition in this experiment (rearing environment: *F*_1,131_ = 0.69, *P* = 0.41; rearing*social: *F*_2,131_ = 1.04, *P* = 0.36; rearing*cactus: *F*_1,131_ = 0.08, *P* = 0.77), and thus we did not include these variables in subsequent analyses to simplify the model. Consequently, our statistical model of interest was:

log(eggs)_csmb_ = µ + τ_c_ + υ_s_ + (τυ)_cs_ + ω_m_ + γ_b_

where µ is the mean response (intercept), τ_c_ is the treatment of cactus quality, υ_s_ is the treatment for social environment, (τυ)_cs_ the interaction of cactus quality and social environment, ω_m_ is female body mass (PC1), and γ_b_ is a random effect of block, γ_b_ ~ N(0, σ^2^). To better interpret the potential treatment effects, we used post hoc multiple comparisons tests (Tukey-Kramer post hoc comparisons). All analyses were conducted using Proc Glimmix in SAS 9.2, by approximating the marginal likelihood using Gauss-Hermite adaptive quadrature with 10 quadrature points [27].

## Results

During the experiment, *Narnia* *femorata* had high survivorship with only one male and one female dying, resulting in data collected from 147 pairs, split approximately equally among experimental groups. Female *N* *. femorata* laid on average 8.3 eggs (range: 0-43) over the seven days in which they were paired with males. Fifty-five females (37% of the total) laid no eggs, and 31 of these females (69% of the females that laid no eggs) were held in pairs on cactus without cactus fruit, the low-quality habitat. Fifteen females (10%) laid 20 or more eggs. We found a significant, positive effect of female body size on the number of eggs produced (Table 1), as expected for female invertebrates.

**Table 1:** Results from a generalized linear mixed model, assuming a Poisson mixture error distribution and a log link function, which tests for effects of social environment, cactus quality, and their interaction on egg numbers.

*Treatment*	*df*	*F-value*	*P-value*
Social environment (social)	2, 135	5.26	0.0063
Cactus quality (cactus)	1, 135	5.30	0.0229
Social × cactus	2, 135	3.84	0.0238
Female size	1, 135	11.82	0.0008

Female size (based on a principal components analysis) was included as a covariate in this model.

**Figure 2:**
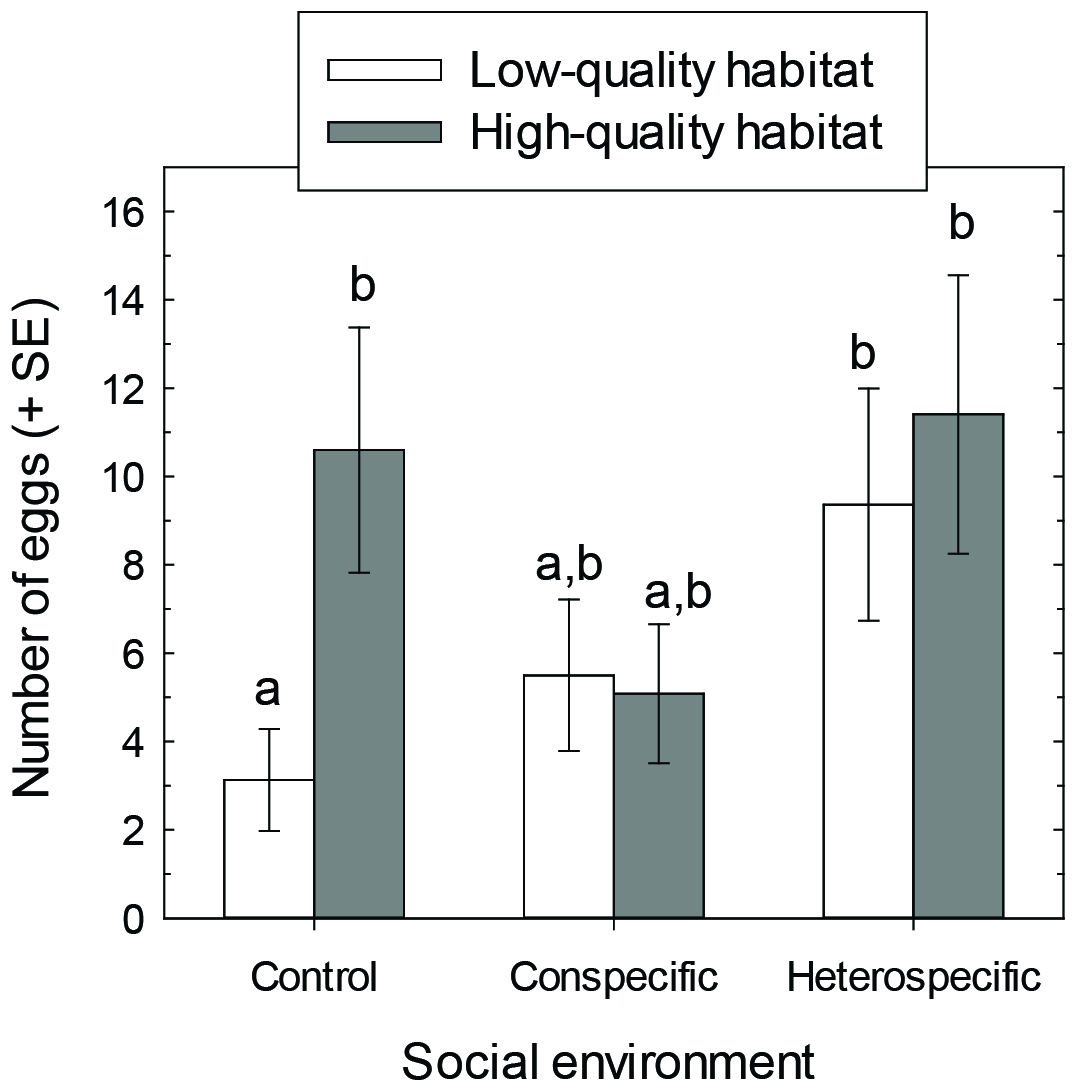
The number of eggs laid(least square means + SE) by *N. femorata* on the basis of social and non-social (resource quality) cues. Resource quality refers to cactus with cactus fruit (high quality) or without cactus fruit (low quality). Egg numbers are adjusted for female size and effects associated with placement in different areas of the greenhouses. Different letters denote significant pair-wise effects (α = 0.05) based on post hoc multiple comparisons (Tukey-Kramer adjusted *P*-values).

The quality of the cactus host was influential to the number of eggs laid. As predicted, on average females laid more eggs when held on the high quality host (cactus with fruit: 8.50 + 1.91 eggs; least squares mean + SE) than on the low quality host (cactus without fruit: 5.44 + 1.34; Table 1). Furthermore, females laid >3x the number of eggs on the high quality host than the low quality host when no juveniles were present (Figure 2).

Not only was cactus quality important, but the social environment also influenced the number of eggs laid. Females laid more eggs in the presence of heterospecific juveniles (10.33 + 2.43) than in the presence of conspecifics (5.76 + 1.52) or alone (5.28 + 1.38). Moreover, the social environment affected the number of eggs laid differently depending upon the quality of the cactus host plant (social × cactus interaction; Table 1 Figure 2). On the low-quality host plant, females laid on average more eggs when heterospecific juveniles were present relative to when they were alone with their mates (post hoc comparison, *P* = 0.04, Figure 2). On the high-quality host plant, females also laid large numbers of eggs with heterospecifics, but the number of eggs laid in the relative absence of social cues was similar (post hoc, *P* = 0.7948).

## Discussion

In the absence of social cues, female *Narnia* *femorata* laid more eggs on the high-quality habitat than the low-quality habitat. However, the presence of conspecifics and heterospecifics eliminated this difference in response (Figure 2). Social cues—be it cues from conspecifics or heterospecifics—appeared to override resource quality in the decision-making by *N* *. femorata* . In other words, resource quality did not significantly influence egg numbers when either conspecifics or heterospecifics were present. Interestingly, *N* *. femorata* appear to lay more eggs in response to heterospecific cues than to conspecific cues. Such direct effects of heterospecific cues on fitness components of individuals have rarely been documented [22].

### Social and non-social cues

Cactus fruits are an important resource for the successful growth and development of *N* *. femorata* , yet they are available only seasonally (Nageon de Lestang & Miller 2009, Gillespie, *unpublished manuscript*), and there are substantial differences in the spatial availability of fruit (C.W. Miller *unpublished data*). Consequently, it is no surprise that female *N* *. femorata* were able to detect the presence of cactus fruit and lay more eggs in a high-quality context of cactus with fruit than the low-quality context without fruit. Interestingly, the presence/absence of cactus fruit was effectively ignored when heterospecific and conspecific juveniles were present.

Social cues can be a very effective means to obtain useful information about the environment; however, using this information is not without costs [28,29]. Social cues are not always accurate predictors of success because other individuals in the environment can make mistakes. Yet, recent studies have revealed that social information can be used preferentially to non-social information [15] and that relying on social information can be the best strategy in certain circumstances [17].

In *N* *. femorata* at least three mechanisms may explain why social cues trump non-social information. First, social cues may simply be a more reliable indicator of habitat quality and future fitness than the presence of a single cactus fruit. Juvenile *N* *. femorata* are frequently found in mixed-age and mixed-species aggregations with *Chelinidia* *vittiger* (Figure 1). It is possible that interactions among juveniles, such as facilitation of feeding or guarding against predators, may be more influential to successful recruitment of offspring than high quality food. Similarly, the quantity of information may matter; the social cue may have more weight because cues from five juveniles overwhelm non-social cues emanating from only one fruit. Second, in natural environments *N* *. femorata* and *C* *. vittiger* juveniles will use areas greater than a single cactus cladode, such that the presence of juveniles may be a more relevant cue (i.e., the cues provide information at a more relevant spatial scale) of habitat quality than the presence of a fruit on a single cladode. Third, the responses to social cues may be dependent upon maximum and minimum limits on egg production. For instance, *C* *. vittiger* juveniles may provide a positive habitat-quality cue when fruit is not present, boosting egg laying to a maximum number. However, when fruit is present, *N* *. femorata* may be unable to lay even more eggs because of a maximal limit to reproductive output. Similarly, egg laying in the presence of conspecific *N* *. femorata* juveniles may be near a minimum, regardless of the presence or absence of cactus fruit. Any benefits or positive cues provided by *N* *. femorata* juveniles may be offset by competitive costs. Even in the absence of mating and/or offspring resources, female *N* *. femorata* and other members of the Hemipteran family Coreidae are constrained to produce several eggs per week, similar to numbers seen in these treatments (Miller 2008, C.W. Miller *unpublished data*).

### Conspecifics versus heterospecifics

Although conspecifics are often emphasized as a primary source of social information, we found that *N* *. femorata* produced more eggs in response to heterospecific cues. This result conflicts with the results from other experiments where responses to heterospecific cues are similar or reduced relative to responses to conspecific cues [30,31]. *Narnia spp*. and *Chelinidea spp*. are commonly found sharing cactus patches in Florida (Figure 1) and elsewhere where their range overlaps (Mexico and the south-western United States). The two species studied here tend to partition foraging on *Opuntia* , with *N* *. femorata* preferring to feed deeply on cactus fruit, whereas *C* *. vittiger* prefers to forage shallowly on cactus *Cladodes* . As a consequence, exploitative competition is not likely severe for these two species. We hypothesize that *N* *. femorata* respond more positively to *C* *. vittiger* cues because such cues may indicate safe resources (due to shared predators) in the absence of intraspecific competitive issues [32,33].

## Conclusions

The role of social cues and the information they encode may have profound, yet generally underappreciated, consequences in biological communities. While early investigations on the use of heterospecific cues focused almost entirely on resident and migrant birds [34,35], our results and other recent work [30,36,37] suggest that the use of heterospecific cues may be common across taxa, even in sub-social insects. In situations where animals do use heterospecific cues, such behaviors can have consequences for individual fitness [22,38], community structure [23,39], and conservation strategies [21]. Furthermore, as environments continue to change through the expansion of invasive species, climate effects altering species distributions, and land-use change, we expect that the use of heterospecific cues will have heightened relevance for community dynamics [40,41].
